# Validity and reliability of the Italian version of the Mild Behavioral Impairment Checklist in cognitively unimpaired and mild cognitive impairment individuals

**DOI:** 10.1177/13872877251380299

**Published:** 2025-10-03

**Authors:** Francesca Remelli, Federico Triolo, Giulia Grande, Maria Giorgia Barbieri, Elena Barbieri, Cristiana Galuppi, Giulia Pampolini, Stefano Volpato, Caterina Trevisan

**Affiliations:** 1 Department of Medical Sciences, 27106 University of Ferrara, Ferrara, Italy; 2 Aging Research Center, Department of Neurobiology, Care Sciences and Society, Karolinska Institutet and Stockholm University, Stockholm, Sweden; 3 Stockholm Gerontology Research Center, Stockholm, Sweden; 4 Orthogeriatric Unit, University Hospital of Ferrara, Ferrara, Italy

**Keywords:** aged, Alzheimer's disease, dementia, loneliness, mild behavioral impairment, Mild Behavioral Impairment Checklist, mild cognitive impairment, neuropsychiatric symptoms, reliability, screening, validity

## Abstract

**Background:**

The Mild Behavioral Impairment Checklist (MBI-C) is a tool for detecting MBI, a neurobehavioral syndrome associated with an increased dementia risk.

**Objective:**

This study aimed to evaluate the reliability and validity of the Italian version of the informant-rated MBI-C in an outpatient sample of dementia-free individuals.

**Methods:**

A cross-sectional study was conducted on 72 older people without dementia (n = 47, mild cognitive impairment; n = 25, cognitively unimpaired). During the visit, physicians administrated the MBI-C and Neuropsychiatric Inventory Questionnaire (NPI-Q) to the informant. Internal consistency of MBI-C was measured by the Cronbach's coefficient alpha and inter-domain correlation coefficients. Diagnostic performance of MBI-C for clinically identified MBI by ISTAART criteria was assessed through ROC analysis, identifying the optimal cut-off based on the Youden Index. Spearman's correlations were used to evaluate the concurrent validity of MBI-C with the NPI-Q, Mini-Mental State Examination (MMSE), Instrumental Activity of Daily Living (IADL) and 3-item UCLA Loneliness Scale.

**Results:**

MBI-C showed high internal consistency ( α = 0.867) and strong inter-domain correlation ( ρ = 0.760 ∼ 0.859, p < 0.001). The Area Under the Curve (AUC) for detecting clinical MBI was 0.937 (95%CI: 0.865–0.972), with an optimal cut-off of 5.5 (sensitivity = 0.849, specificity = 0.876). The MBI-C total score strongly correlated with the NPI-Q total score ( ρ = 0.820, p < 0.001). Only the MBI-C total score significantly correlated with the 3-item UCLA ( ρ = 0.236, p = 0.046); no significant correlations were found with MMSE and IADL scores.

**Conclusions:**

The Italian version of MBI-C demonstrated strong reliability, validity, and diagnostic performance. Therefore, MBI-C may be a suitable tool for assessing behavioral symptoms in dementia-free individuals.

## Introduction

Neuropsychiatric symptoms (NPS) are increasingly recognized as core features of the neurodegenerative process, often emerging in the early stages of dementia.^1,2^ Although NPS have been traditionally considered secondary to cognitive decline, a growing body of research suggests that their onset may occur several years before overt cognitive impairment.^3–5^ The framework of mild behavioral impairment (MBI) has been introduced to characterize persistent and impactful NPS that emerge in late-life in individuals without dementia.^6,7^ Previous studies have identified MBI as a neurobehavioral syndrome with potential prognostic value in the progression from mild cognitive impairment (MCI) to dementia, capturing early behavioral symptoms specific to neurocognitive disorder.^1,8–11^ The prevalence of MBI was reported extremely high in the older population, ranging from 27.6% to 43.1% among cognitively unimpaired individuals,^1,12^ and increasing to nearly 60% in those with MCI.^13^

Nowadays, in clinical practice, the Neuropsychiatric Inventory Questionnaire (NPI-Q) is the most used screening tool for assessing NPS. The NPI-Q comprises twelve specific items and was initially designed for individuals affected by dementia.^14^ Although the NPI-Q has been previously employed for detecting MBI,^1,15,16^ it might fail to detect subtle yet persistent symptoms that can characterize MBI, potentially leading to underestimating this condition. In light of this potential limitation, the Mild Behavioral Impairment Checklist (MBI-C) has been proposed as a tool for neuropsychiatric assessment for individuals free from dementia.^17^ Developed in accordance with the ISTAART criteria, this instrument consists of 34 items that map onto the five domains of MBI (i.e., decreased motivation, affective dysregulation, impulse dyscontrol, social inappropriateness, and abnormal perception or thought content) and has been translated into 16 languages.^18^ The MBI-C offers a practical and standardized screening tool—quick, easy to administer, and cost-effective.^17^

Although evidence supporting the clinical utility of MBI is increasing, it remains largely underrecognized in clinical practice, where routine assessments focus primarily on cognitive testing. Assessing the psychometric properties of the MBI-C in clinical settings could facilitate its broader adoption, especially considering the urgent need for improved early detection of neurodegenerative diseases. However, to date, the Italian version of the MBI-C has not yet been validated.

The aim of this study is to evaluate the reliability and validity of the Italian version of the informant-rated MBI-C in a clinical sample of dementia-free individuals.

## Methods

### Study design and participants

This cross-sectional study was conducted at the geriatrics outpatient Memory Clinic of the University Hospital of Ferrara (Italy). Individuals referred to the service due to cognitive or behavioral complaints between 1 March 2024 and 4 April 2025 were consecutively enrolled. The inclusion criteria were: 1) age 65 years or older, 2) native Italian speakers, 3) absence of a dementia diagnosis, either retrieved through medical assessment or information from the caregiver, 4) presence of a informant/caregiver. From the initial sample of 88 patients, 16 had a previous diagnosis of dementia and were excluded. The analytical sample included 72 participants.

During the medical visit, all participants underwent a clinical interview that included cognitive and neuropsychiatric evaluations, assessment of loneliness, as well as socio-demographic information, physical performance, and medical and pharmacological histories. Clinical diagnoses of MCI and dementia were based on the assessment of cognitive and physical performance and made according to the Diagnostic and Statistical Manual of Mental Disorders, Fifth Edition (DSM-5).^19^ Specifically, MCI was diagnosed by the geriatrician during the medical visit based on evidence of cognitive impairment, indicated by a Mini-Mental State Examination (MMSE)^20^ score less than 27, with no impact on autonomy in Basic Activities of Daily Living (BADL).^21^ Among those free from dementia, when MCI was absent, participants were classified as cognitively unimpaired. The study protocol was approved by the local Ethics Committee (241/2024/Oss/AOUFe). All participants provided written informed consent.

### Neuropsychiatric assessment

At the end of the medical examination, NPS were assessed through MBI-C and NPI-Q, both administered by the geriatrician to the informant.

Concerning the MBI-C, we used the Italian version previously translated by Elefante et al.^18,22^ Based on their work, first, the English version of MBI-C was independently translated into Italian by two researchers. Second, following the standard back-translation procedure, the Italian version was translated back into English and its main author (Ismail Z.)^17^ established the semantic equivalence between the Italian version and the original English MBI-C.^22^ MBI-C assesses the presence of 34 items over the past 6 months, grouped into 5 symptom domains: 6 items for decreased motivation, 6 items for affective dysregulation, 12 items for impulse dyscontrol, 5 items for social inappropriateness, and 5 items for abnormal perception or thought content.^17^

NPI-Q includes 12 items that assess the presence of the following symptoms in the past month: delusions, hallucinations, agitation/aggression, depression/dysphoria, anxiety, elation/euphoria, apathy/indifference, disinhibition, irritability/lability, motor disturbance, nighttime behaviors, and appetite/eating.^14,23,24^

In both the MBI-C and NPI-Q, symptom severity is assessed on a 3-level scale: 1 (Mild) for NPS observable but without significant change from the longstanding pattern of behavior, 2 (Moderate) for NPS significant but without major change, and 3 (Severe) for NPS very marked or dramatic change. The total scores of MBI-C and NPI-Q are calculated based on the sum of the single NPS severities; 0 points were given for absent symptoms.

To explore the correlation between MBI domains and NPI-Q, we used a previous published operationalization algorithm to map NPI-Q items into five MBI domains.^1^

### Mild behavioral impairment

MBI was clinically defined during the medical interview according to the ISTAART criteria.^7^ The physician who performed the diagnosis was blinded to MBI-C score results. One or more NPS were considered indicative of the presence of MBI when they represented a clear change from the patient's usual behavior, and were persistent for at least 6 months. The behavioral changes were reported in at least one of the following five neuropsychiatric domains: 1) decreased motivation, 2) affective dysregulation, 3) impulse dyscontrol, 4) social inappropriateness, and 5) abnormal perception or thought content. As suggested by the ISTAART criteria, NPS had to be sufficiently severe to cause at least minimal reported impairment in social functioning, without affecting independence in daily life activities.^7^ No current psychiatric disorders, somatic conditions, or ongoing pharmacological terapies justified the onset of NPS when MBI was detected. Moreover, individuals with MBI did not meet the diagnostic criteria for dementia, while MCI could be simultaneously present alongside MBI.^7^

### Loneliness assessment

The 3-item UCLA Loneliness Scale was used to examine loneliness.^25,26^ This is a quick and simple interviewer-administered questionnaire, derived from the Revised UCLA Loneliness Scale,^27^ and consists of three questions: *1. How often do you feel that you lack companionship? 2. How often do you feel left out? 3. How often do you feel isolated from others?*

Based on the frequency of these feelings, the response options are rated as 1 for “hardly ever”, 2 for “some of the time”, and 3 for “often”. The total score, obtained by summing all items, allows for an indirect assessment of loneliness severity, where higher scores indicate greater levels of loneliness.^25,28^ The scale was administered by the geriatrician to the patient during the medical examination.

### Others measures

For each participant, the following data were collected: socio-demographic information (age, sex, education), physical performance (BADL and Instrumental Activities of Daily Living [IADL]),^21,29,30^ main chronic diseases (including the presence of arterial hypertension, ischemic heart disease, atrial fibrillation, chronic heart failure, previous stroke, diabetes, Chronic Obstructive Pulmonary Disease, chronic psychiatric diseases), and pharmacological therapy (number of medications, anticholinergic burden by the ACB score,^31^ and type of psychoactive medication, if taken, i.e., antidepressants, benzodiazepines, and antipsychotics).

The MMSE was used to assess the global cognitive performance.^32^

### Statistical analysis

Participants’ characteristics were compared based on the presence of MCI using the Student's t-test, Kruskal-Wallis test, Chi-squared test, or Fisher's exact test, as appropriate. Categorical variables were presented as counts and proportions, while continuous variables as means and standard deviations. To assess the internal consistency of the MBI-C in the whole sample, Cronbach's coefficient alpha and inter-domain correlation coefficients were calculated. The diagnostic performance of MBI-C for clinical MBI, diagnosed according to ISTAART criteria, was assessed through the ROC analysis, identifying the optimal cut-off based on the Youden Index. Sensitivity, specificity, Positive Predictive Value (PPV), Negative Predictive Value (NPV) and the Youden Index were further calculated at the commonly used cut-off score of 6.5 to evaluate the MBI-C's discriminatory ability.^33–35^ In addition, the DeLong test was used to compare the Area Under the Curves (AUCs) of the MBI-C and NPI-Q for predicting clinical MBI diagnosis. Concurrent validity was evaluated using Spearman's correlation analysis, examining the association between the MBI-C total score and the total scores of NPI-Q, MMSE, IADL, and 3-item UCLA Loneliness Scale. The relationship between the MBI-C and NPI-Q was further explored by analyzing correlations between individual MBI and NPI domains. The strength of the association was considered strong when the rho ( ρ ) value was ≥0.6.

To ensure that the findings were not driven by the inclusion of participants with chronic psychiatric disorders, we performed a sensitivity analysis excluding individuals with a chronic antipsychotic use (N = 2) and those endorsed delusional beliefs and hallucinations (N = 8).

An exploratory factor analysis was conducted to examine the latent factor structure of the Italian version of the MBI-C, using maximum likelihood extraction and oblimin rotation. The Kaiser-Meyer-Olkin (KMO) test and Bartlett's test of sphericity were performed to assess the suitability of the data for exploratory factor analysis. Based on both the scree plot and parallel analysis, the suggested number of factors to retain was five, consistent with the theoretical model. Factor loadings and communalities (h²) were calculated for each item. Items were considered to load substantially on a factor if their loading was ≥0.40.^36,37^

Statistical analyses were conducted using R statistical software (version 4.4.3), with statistical significance at p value <0.05.

## Results

### Characteristics of study participants

The mean age of the 72 participants was 78.3 years; 56.9% were female, and the mean years of education was 7.8. The participants had a mean MMSE of 24.8, and, on average, were self-sufficient in 5.3 out of 6 BADL. Overall, the mean total score of MBI-C was 7.5, while for NPI-Q it was 3.9. The prevalence of MBI clinically detected according to ISTAART criteria was 62.7% (n = 42), and it co-occurred with MCI in 38.9% (n = 28) of cases. When comparing participants cognitively unimpaired (n = 25, 37.3%) and those with MCI (n = 47, 62.7%) (Table 1), there was no significant differences in total scores of MBI-C and NPI-Q, while the mean MMSE was significantly lower in those with MCI.

**Table 1.** Participants’ characteristics according to the presence of MCI.

	Overall (N = 72)	Cognitively unimpaired (N = 25)	MCI (N = 47)	p
Age	78.3 (5.1)	79.5 (4.6)	77.7 (5.3)	0.155
Females, n (%)	41 (56.9)	12 (48.0)	29 (61.7)	0.385
Education (y)	7.8 (3.5)	8.4 (3.7)	7.5 (3.4)	0.295
BADL	5.3 (0.9)	5.1 (0.9)	5.4 (0.9)	0.076
IADL				
*Females*	6.0 (2.4)	5.4 (2.4)	6.3 (2.3)	0.312
Males*	4.2 (1.3)	4.0 (1.3)	4.3 (1.3)	0.499
Chronic diseases, n (%)				
*Arterial hypertension*	53 (75.0)	22 (88.0)	32 (68.9)	0.116
Chronic heart diseases**	23 (31.9)	8 (32.0)	15 (31.9)	1.000
*Previous stroke*	5 (6.9)	4 (16.0)	1 (2.2)	0.086
*Diabetes*	16 (22.2)	6 (24.0)	10 (21.3)	1.000
*CODP*	6 (8.3)	3 (12.0)	3 (6.4)	0.709
*Previous psychiatric history*	13 (18.1)	5 (20.0)	8 (17.0)	1.000
No. of drugs	6 (3.4)	7.1 (3.5)	5.4 (3.2)	**0**.**034**
Use of psychoactive drugs n (%)	23 (31.9)	11 (44.0)	12 (25.5)	0.182
*Antidepressants*	14 (19.4)	5 (20.0)	9 (19.1)	1.000
*Benzodiazepines*	12 (16.7)	9 (36.0)	3 (6.4)	**0**.**004**
*Antipsychotics*	2 (2.8)	1 (4.0)	1 (2.1)	1.000
Anticholinergic burden***	1.3 (1.2)	1.2 (1.2)	1.4 (1.1)	0.659
MMSE total score	24.8 (3.7)	27.2 (2.0)	23.5 (3.8)	**<0**.**001**
NPI-Q total score	3.9 (3.9)	3.9 (3.8)	4.0 (3.9)	0.969
MBI-C total score	7.5 (7.8)	8.2 (8.7)	7.1 (7.3)	0.604
*Decreased motivation score*	2.3 (3.6)	2.5 (4.5)	2.2 (3.0)	0.744
*Affective dysregulation score*	2.3 (3.5)	2.9 (4.3)	2.0 (2.9)	0.318
*Impulse dyscontrol score*	1.9 (2.7)	2.1 (2.5)	1.8 (2.9)	0.692
*Social inappropriateness score*	0.6 (1.6)	0.7 (1.6)	0.6 (1.6)	0.828
*Abnormal perception score*	0.4 (1.5)	0.1 (0.3)	0.6 (1.9)	0.190
3-item UCLA total score	1.2 (1.7)	1.3 (2.0)	1.1 (1.5)	0.609

If not specified, tables report mean (standard deviation).

MCI: mild cognitive impairment; BADL: Basic Activities of Daily Living; IADL: Instrumental Activities of Daily Living; COPD: chronic obstructive pulmonary disease; MMSE: Mini-Mental State Examination; NPI-Q: Neuropsychiatric Inventory Questionnaire; MBI-C: Mild Behavioral Impairment Checklist; 3-item UCLA: 3-item UCLA Loneliness Scale.

*IADL score was calculated out of 8 for females, and out of 5 for males.

**Chronic heart diseases included either ischemic heart disease, heart failure, or atrial fibrillation.

***Anticholinergic burden was calculated by the ACB score.

**Figure 1.**
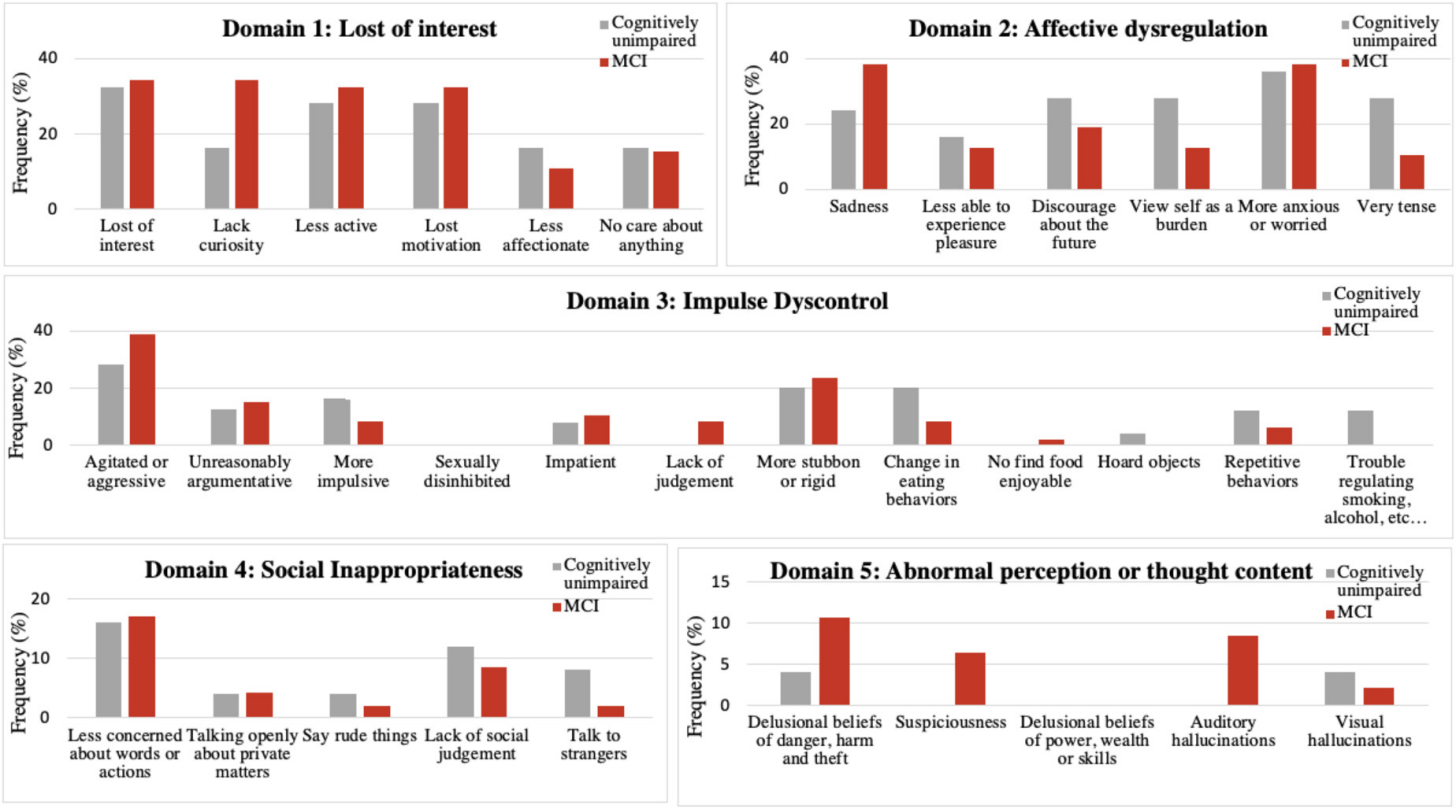
Frequency of NPS detected by MBI-C in the study sample according to the presence of MCI.

As shown in Figure 1, neuropsychiatric screening by the MBI-C revealed that the most common identified symptoms were those related to Domain 1 (i.e., lost of interest −33.3%, less active – 30.6%, and lost motivation – 30.6%) and Domain 2 (i.e., sadness – 33.3%, and more anxious or worried – 37.5%). Agitation, assessed in the Domain 3, was also frequently reported (34.7%). Unlike all other investigated symptoms, sexual disinhibition and delusional beliefs of power-wealth-skills were not detected in our sample. When comparing the two study groups, the cognitively unimpaired and MCI groups did not exhibit statistically significant differences in the frequency of MBI symptoms as assessed by the MBI-C.

### Internal consistency of the MBI-C

The Cronbach's coefficient alpha of MBI-C was 0.867. Moreover, the MBI-C total score was positively correlated with the five MBI domain scores ( ρ = 0.760 ∼ 0.859, p < 0.001). As reported in Table 2, all inter-domain correlation coefficients among the MBI domains were statistically significant. Strong correlations were observed between decreased motivation and affective dysregulation ( ρ = 0.618, p < 0.001), social inappropriateness and impulse dyscontrol ( ρ = 0.718, p < 0.001), abnormal perception or thought content and both impulse dyscontrol and social inappropriateness ( ρ = 0.782, p < 0.001 and ρ = 0.874, p < 0.001, respectively).

**Table 2.** Inter-domain correlation coefficients of the five domains and the total MBI-C score.

	Decreased motivation	Affective dysregulation	Impulse dyscontrol	Social inappropria-teness	Abnormal perception or thoughts	Total MBI-C score
Decreased motivation	-					
Affective dysregulation	0.618	-				
Impulse dyscontrol	0.454	0.574	-			
Social inappropriateness	0.373	0.385	0.718	-		
Abnormal perception or thought content	0.435	0.453	0.782	0.874	-	
Total MBI-C score	0.760	0.801	0.859	0.771	0.827	-

MBI-C: Mild Behavioral Impairment Checklist; NPI-Q: Neuropsychiatric Inventory Questionnaire.

In the table are reported the significant results.

### Diagnostic performance of the MBI-C

**Figure 2.**
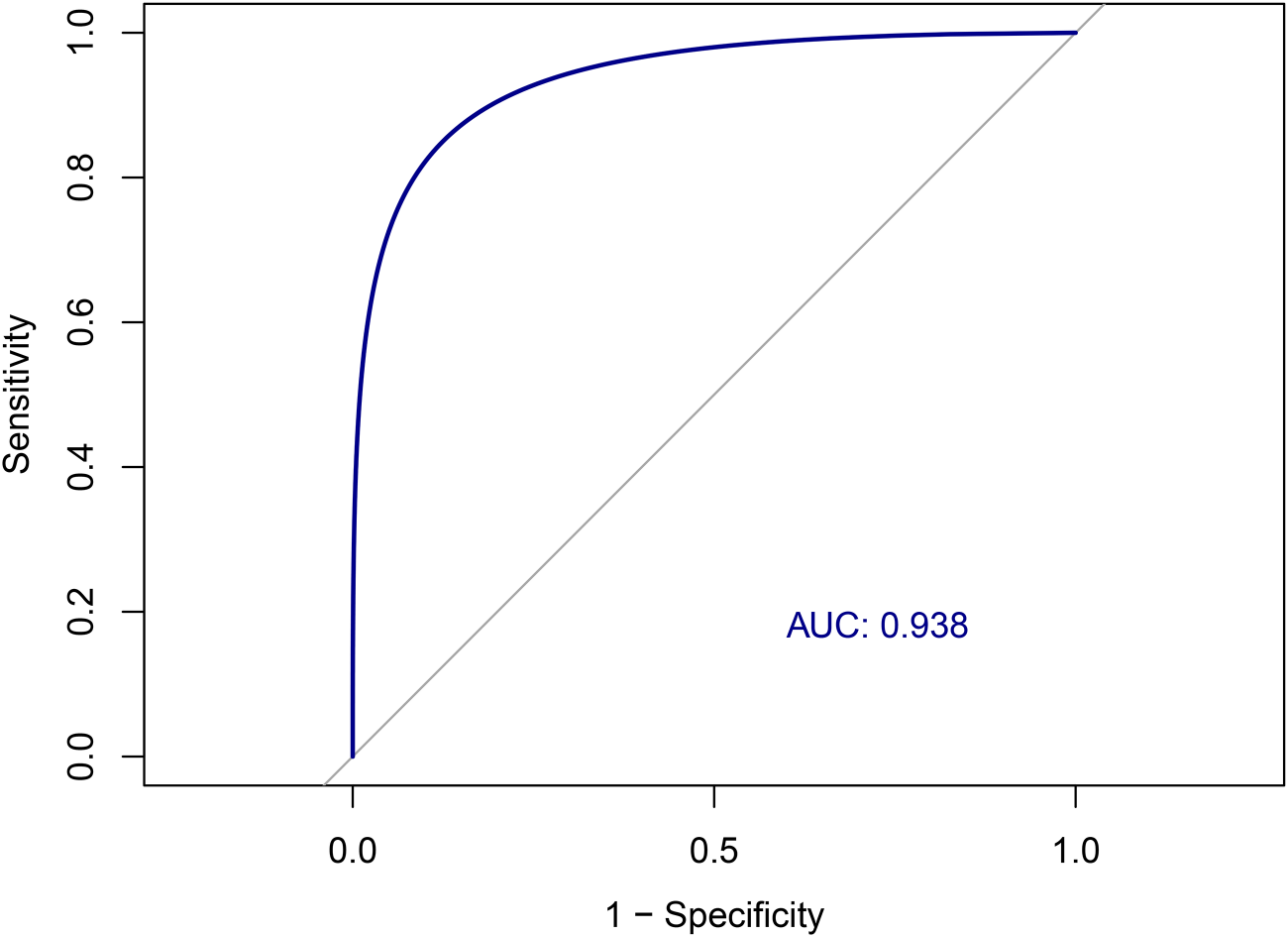
ROC curve for the detection of clinical MBI by ISTAART criteria using the the MBI-C.

The Area Under the Curve (AUC) for the prediction of clinical MBI diagnosis, made according to ISTAART criteria, was 0.937 (95%CI: 0.865–0.972) using the MBI-C (Figure 2). According to the Youden Index, the optimal cut-off for MBI-C on our sample was 5.5, yielding a sensitivity of 0.849, specificity of 0.876, a PPV and NPV of 0.969 and 0.725, respectively, and a Youden Index of 0.725. When applying the commonly used cut-off of 6.5 for MBI, the MBI-C demonstrated a sensitivity of 0.691, a specificity of 0.967, a PPV and NPV of 0.967 and 0.690, respectively, corresponding to a Youden Index of 0.657.

Repeating the analysis using the NPI-Q, the AUC was 0.846 (95%CI: 0.743–0.928), which was significantly lower than that obtained with the MBI-C (DeLong test: p = 0.010).

### Concurrent validity of the MBI-C

The MBI-C total score strongly correlated with the NPI-Q total score ( ρ = 0.820, p < 0.001). The individual MBI domain scores were also significantly correlated with the NPI-Q total score ( ρ = 0.327 ∼ 0.569, p < 0.001), while the correlations with the individual NPI-Q domain scores reached the statistical significance only in specific domains (Table 3).

**Table 3.** Correlations between MBI-C and NPI-Q.

		NPI-Q					
		Apathy	Mood symptoms	Agitation	Disinhibition	Psychosis	Total score
MBI-C	Decreased motivation	0.680	0.324	0.308	ns	ns	**0**.**471**
	Affective dysregulation	ns	0.705	ns	ns	0.322	**0**.**569**
	Impulse dyscontrol	0.223	ns	0.765	0.216	0.277	**0**.**501**
	Social inappropriateness	ns	ns	0.351	0.389	0.227	**0**.**327**
	Abnormal perception or thoughts	ns	0.279	0.244	0.265	0.940	**0**.**455**
	Total score	**0.466**	**0**.**603**	**0**.**518**	**0**.**230**	**0**.**386**	**0**.**820**

MBI-C: Mild Behavioral Impairment Checklist; NPI-Q: Neuropsychiatric Inventory Questionnaire; ns, not significant (p ≥ 0.05).

In the table are reported only the significant ρ .

Exploring the correlation between MBI-C and other investigated scales, neither the total score of MBI-C nor the total score of NPI-Q was significantly correlated with MMSE ( ρ = 0.033, p = 0.782 and ρ = 0.006, p = 0.961, respectively) or IADL ( ρ = −0.153, p = 0.200 and ρ = −0.167, p = 0.160, respectively). Only the MBI-C total score was significantly and positively correlated with the 3-item UCLA Loneliness Scale (MBI-C: ρ = 0.236, p = 0.046; NPI-Q: ρ = 0.104, p = 0.386).

### Sensitivity analyses

After excluding participants with chronic antipsychotic use (N = 2) and those who endorsed delusional beliefs and hallucinations (N = 8), the overall results remained consistent (*data not shown*).

### Exploratory factor analysis

Items with no response (i.e., sexual disinhibition and delusional beliefs of power-wealth-skills) were removed from the analysis. The suitability of the data for exploratory factor analysis was supported by a KMO measure of sampling adequacy of 0.612 and a significant Bartlett's test of sphericity (χ² = 1836.822, df = 528, p < 0.001). As shown in Table 4, the factor structure of the Italian version of the MBI-C corresponded well with the original domains for Domain 1 (Loss of Interest) and Domain 2 (Affective Dysregulation), showed minor discrepancies in Domain 3 (Impulse dyscontrol), and demonstrated more substantial differences in Domains 4 (Social inappropriateness) and 5 (Abnormal perception or thought content).

**Table 4.** Exploratory factor analysis of the Italian version of MBI-C.

		Italian MBI-C					
Original MBI-C	Items	Factor 1	Factor 2	Factor 3	Factor 4	Factor 5	h^2^
Domain 1: Lost of interest	Lost of interest	0.71					0.586
	Lack curiosity	0.78					0.603
	Less active	0.87					0.820
	Lost motivation	0.74					0.533
	Less affectionate	0.78					0.651
	No care about anything	0.77					0.682
Domain 2: Affective dysregulation	Sadness		0.69				0.545
	Less able to experience pleasure		0.52				0.507
	Discourage about the future		0.60				0.538
	View self as a burden		0.57				0.667
	More anxious or worried		0.77				0.594
	Very tense		0.83				0.734
Domain 3: Impulse dyscontrol	Agitated or aggressive			0.66			0.511
	Unreasonably argumentative			0.82			0.680
	More impulsive			0.63			0.537
	Sexually disinhibited						
	Impatient			0.25			0.111
	Lack of judgement			0.45			0.212
	More stubbon or rigid			0.70			0.559
	Change in eating behaviors		0.41				0.226
	No find food enjoyable	0.05					0.014
	Hoard objects	0.40					0.168
	Repetitive behaviors		0.54				0.362
	Trouble regulating smoking, alcohol, etc.					0.78	0.710
Domain 4: Social inappropriateness	Less concerned about words or actions			0.57			0.506
	Talking openly about private matters				0.76		0.683
	Say rude things			0.20			0.049
	Lack of social judgement			0.52			0.614
	Talk to strangers				0.96		0.995
Domain 5: Abnormal perception or thought content	Delusional beliefs of danger, harm and theft			0.76			0.752
	Suspiciousness			0.75			0.686
	Delusional beliefs of power, wealth or skills						
	Auditory hallucinations			0.82			0.856
	Visual hallucinations				0.67		0.669

## Discussion

This study demonstrates the reliability and validity of the Italian version of MBI-C in a population without dementia referred to a outpatient Memory Clinic. Except for sexual disinhibition and delusional beliefs of power, wealth, and skills, all other NPS investigated using the MBI-C were detected in the sample, with no substantial differences between cognitively unimpaired individuals and those with MCI. Beyond high internal consistency, the MBI-C showed excellent discriminatory performance for MBI defined according to ISTAART criteria, surpassing that obtained with NPI-Q, with an optimal cut-off score of 5.5 points. Concurrent validation was supported by a significant correlation with the NPI-Q, which is traditionally used to assess NPS in individuals living with dementia. Notably, unlike the NPI-Q, the MBI-C uniquely correlated with the severity of loneliness perceived by the patient.

No significant differences in the prevalence of NPS, screened by the MBI-C, were found between cognitively unimpaired and MCI individuals. These findings contrast with previous studies, which reported a higher prevalence of NPS in individuals with MCI.^12,15^ However, beyond low statistical power, this discrepancy may be attributed to the heightened sensitivity of the MBI-C, compared with NPI-Q, in detecting subtle symptoms that could already be present in cognitively unimpaired participants. Additionally, a potential underestimation of MCI prevalence in our sample cannot be excluded, as the cognitive assessment was based solely on the MMSE, which may not have intercepted certain cognitive deficits, such as executive dysfunction.^32,38,39^ Regarding the individual NPS, sexual disinhibition and delusional beliefs related to power, wealth, and skills were not detected among the enrolled individuals. In addition to potential cultural factors that may lead both patients and informants to underreport certain NPS in older adults, these behaviors may be more frequently observed after the onset of dementia, particularly in specific subtypes such as frontotemporal dementia, which may not have been represented in our sample.^40^

The internal consistency of the Italian version of MBI-C was excellent, with a Cronbach's alpha of 0.867. This is comparable to the original English version (alpha = 0.87)^17^ and other translations,^41–43^ indicating that the translation into Italian has not affected the instrument's reliability. Furthermore, the MBI-C total score showed strong positive correlations with each of the five behavioral domains, and significant inter-domain relationships were observed. Notably, some correlations between certain domains were stronger than others. Specifically, the following domains were particularly interrelated: decreased motivation with affective dysregulation, social inappropriateness with impulse dyscontrol, and abnormal thoughts with both impulse dyscontrol and social inappropriateness. These findings align with the nature of the investigated symptoms, as decreased motivation and affective dysregulation are linked to emotions and mood alterations, while the other three domains are more connected to behavioral and psychotic dimensions. Furthermore, these results may also reflect the likely localization of the underlying neuropathological changes, with mood symptoms or apathy more commonly associated with temporal lobe involvement,^44,45^ and disinhibition and psychosis linked to a greater frontal lobe pathology,^46,47^ depending on the dementia subtype at onset. Nevertheless, although there is solid evidence about MCI progression to dementia, which may be due to various underlying neurodegenerative and non-neurodegenerative processes,^48^ limited research is currently available on the course of MBI. Therefore, further studies with a longitudinal design and biological data (e.g., biomarkers of neurodegeneration and neuroinflammation) are needed for a deeper understanding of the prognostic role of MBI in cognitively unimpaired individuals.

The diagnostic performance of the Italian version of MBI-C was excellent, with an AUC of 0.937 for detecting clinically diagnosed MBI, which was significantly higher than that observed with the NPI-Q. While the commonly used cut-off score of 6.5 offered high specificity (0.967) but moderate sensitivity (0.691), the Youden Index analysis suggested that a lower cut-off of 5.5 optimizes diagnostic performance (sensitivity = 0.849; specificity = 0.876). Although the most commonly used cut-off for MCI is 6.5,^33–35^ our findings are consistent with previous studies that fixed 5.5 as the threshold to have the best discriminative performance.^42,49,50^ Further studies are needed to validate the cut-off of 5.5 in broader populations or clinical subgroups.

Regarding concurrent validity, the Italian version of the MBI-C total score was strongly correlated with the NPI-Q total score. Although the MBI-C and NPI-Q were designed for different contexts, the MBI-C for early sustained symptoms preceding dementia diagnosis, and the NPI-Q for neuropsychiatric symptoms in dementia, their convergence supports the MBI-C's capacity to capture meaningful changes in neuropsychiatric symptoms. Furthermore, the correlations between MBI-C domains and NPI-Q scores align with previous studies, supporting the view that the MBI-C reflects a spectrum of behavioral symptoms relevant across cognitive stages.^43,51^ Additionally, the different reference periods for the symptoms assessed by the MBI-C and NPI-Q may have influenced the observed correlation between them. While the MBI-C assesses NPS over a 6-month period, capturing more persistent or cumulative behavioral changes, the NPI-Q evaluates symptoms within a shorter, 1-month timeframe, which may reflect more transient or recent manifestations. This discrepancy could lead to an overestimation of MBI diagnosis when using the NPI-Q instead of the MBI-C in free-dementia individuals, potentially including NPS that are not indicative of an underlying neurodegenerative process at onset, but instead of transient organic or functional disorders.

Importantly, this study also examined the relationship between behavioral symptoms and other clinical variables. Neither the MBI-C nor NPI-Q scores were significantly correlated with MMSE or IADL scores, suggesting, beyond the intrinsic definition of MBI, the behavioral changes assessed by the MBI-C are not merely secondary to cognitive decline or functional loss.^5–7^ However, unlike the NPI-Q total score, the MBI-C was significantly correlated with the 3-item UCLA Loneliness Scale. A previous study conducted in a Japanese cohort to validate the MBI-C also reported a consistent association between these two scales.^42^ The MBI-C detects loneliness severity more effectively than the NPI-Q, highlighting a key issue in the at-risk dementia population. Although the reliability of self-rated MBI-C needs further investigation, the informant-rated MBI-C appears to be particularly suitable for the neuropsychiatric assessment of dementia-free individuals, as it captures several behavioral aspects, such as loneliness, which is widely recognized as being associated with a higher likelihood of progression toward dementia.^52–54^ Although no longitudinal studies have yet explored the interaction between MBI and loneliness, it is plausible to hypothesize that they play a synergistic role in dementia development. Reducing loneliness may help mitigate the progression from MBI to dementia, but intervention studies with long-term follow-up are needed to confirm this hypothesis.

Exploratory factor analysis revealed that most of the factors, specifically those corresponding to Domain 1 (Loss of Interest), Domain 2 (Affective Dysregulation), and Domain 3 (Impulse dyscontrol), were consistent with the original MBI-C structure, whereas items previously incorporated into Domains 4 (Social inappropriateness) and 5 (Abnormal perception or thought content) demonstrated substantial differences. Specifically, the fifth factor consisted of only one item (*Trouble regulating smoking, alcohol, etc.*), while the original Impulse Dyscontrol, Social Inappropriateness, and Abnormal Perception or Thought Content domains merged into two factors. This pattern is likely driven by the low prevalence of NPS belonging to Domains 4 and 5 in our sample, as well as the clinical overlap of symptoms in Domains 3 and 4. Moreover, the items *Change in eating behaviors* and *Repetitive behaviors* loaded onto the Affective Dysregulation factor, which may be compatible with mood symptoms related to depression and anxiety. Notably, the item *Hoard objects* loaded onto the Loss of Motivation factor but demonstrated very low communality. While our findings do not align with those reported by Creese et al. in cognitively intact older adults,^51^ the results from Xu et al., who validated the Chinese version of the MBI-C in a population with MCI or mild AD, also revealed some discrepancies compared with the original MBI-C.^43^ Although our sample differs from those enrolled in these two mentioned studies,^43,51^ further investigations will be necessary to clarify this issue.

A key strength of this study is that it represents the first attempt to validate the MBI-C in the Italian population, supporting its potential applicability in clinical practice. Moreover, patients were consecutively recruited and underwent a comprehensive medical examination. However, some limitations should be discussed when interpreting these findings. First, the timing of NPS onset, assessed by the MBI-C and NPI-Q, differed between the two tools (six months and one month, respectively), which may have introduced variability in symptom reporting and reduced the precision of their direct comparison. Nevertheless, both scales were administered to the informant, ensuring a high degree of consistency in the reported information. Second, beyond the BADL and IADL assessments, individuals were classified as MCI only based on MMSE scores, which may have led to an underestimation of subtle cognitive deficits, particularly executive dysfunctions, with possible misclassification bias. Nevertheless, the MCI diagnosis was always confirmed by a geriatrician during the medical visit and was used exclusively to describe the sample, not in any inferential analyses. Further validation studies on cognitively unimpaired individuals are needed to understand the clinical applicability of the MBI-C in a non-specialist setting.

### Conclusions

The Italian version of the MBI-C is a reliable and valid tool for assessing behavioral changes in individuals without dementia. Its strong internal consistency, diagnostic accuracy, and correlation with established measures suggest it is well-suited for clinical practice, facilitating the early identification of individuals at risk for dementia. Notably, the MBI-C's ability to capture the severity of loneliness, often overlooked by traditional tools, enhances its relevance in neuropsychiatric assessment of dementia-free individuals.
